# Reconstruction of the human nipple–areolar complex: a tissue engineering approach

**DOI:** 10.3389/fbioe.2023.1295075

**Published:** 2024-02-15

**Authors:** Louis Maistriaux, Vincent Foulon, Lies Fievé, Daela Xhema, Robin Evrard, Julie Manon, Maude Coyette, Caroline Bouzin, Yves Poumay, Pierre Gianello, Catherine Behets, Benoît Lengelé

**Affiliations:** ^1^ Pole of Morphology (MORF), Institute of Experimental and Clinical Research (IREC), UCLouvain, Brussels, Belgium; ^2^ Pole of Experimental Surgery and Transplantation (CHEX), Institute of Experimental and Clinical Research (IREC), UCLouvain, Brussels, Belgium; ^3^ Department of Plastic and Reconstructive Surgery, Cliniques Universitaires Saint-Luc, Brussels, Belgium; ^4^ IREC Imaging Platform (2IP), Institute of Experimental and Clinical Research (IREC), UCLouvain, Brussels, Belgium; ^5^ Research Unit for Molecular Physiology (URPhyM), Department of Medicine, Namur Research Institute for Life Sciences (NARILIS), UNamur, Namur, Belgium

**Keywords:** nipple–areolar complex, nipple–areolar complex reconstruction, tissue engineering, decellularization, recellularization, extracellular matrix, ECM, reconstructive surgery

## Abstract

**Introduction:** Nipple–areolar complex (NAC) reconstruction after breast cancer surgery is challenging and does not always provide optimal long-term esthetic results. Therefore, generating a NAC using tissue engineering techniques, such as a decellularization–recellularization process, is an alternative option to recreate a specific 3D NAC morphological unit, which is then covered with an *in vitro* regenerated epidermis and, thereafter, skin-grafted on the reconstructed breast.

**Materials and methods:** Human NACs were harvested from cadaveric donors and decellularized using sequential detergent baths. Cellular clearance and extracellular matrix (ECM) preservation were analyzed by histology, as well as by DNA, ECM proteins, growth factors, and residual sodium dodecyl sulfate (SDS) quantification. *In vivo* biocompatibility was evaluated 30 days after the subcutaneous implantation of native and decellularized human NACs in rats. *In vitro* scaffold cytocompatibility was assessed by static seeding of human fibroblasts on their hypodermal side for 7 days, while human keratinocytes were seeded on the scaffold epidermal side for 10 days by using the reconstructed human epidermis (RHE) technique to investigate the regeneration of a new epidermis.

**Results:** The decellularized NAC showed a preserved 3D morphology and appeared white. After decellularization, a DNA reduction of 98.3% and the absence of nuclear and HLA staining in histological sections confirmed complete cellular clearance. The ECM architecture and main ECM proteins were preserved, associated with the detection and decrease in growth factors, while a very low amount of residual SDS was detected after decellularization. The decellularized scaffolds were *in vivo* biocompatible, fully revascularized, and did not induce the production of rat anti-human antibodies after 30 days of subcutaneous implantation. Scaffold *in vitro* cytocompatibility was confirmed by the increasing proliferation of seeded human fibroblasts during 7 days of culture, associated with a high number of living cells and a similar viability compared to the control cells after 7 days of static culture. Moreover, the RHE technique allowed us to recreate a keratinized pluristratified epithelium after 10 days of culture.

**Conclusion:** Tissue engineering allowed us to create an acellular and biocompatible NAC with a preserved morphology, microarchitecture, and matrix proteins while maintaining their cell growth potential and ability to regenerate the skin epidermis. Thus, tissue engineering could provide a novel alternative to personalized and natural NAC reconstruction.

## 1 Introduction

Breast cancer is the most common cancer worldwide, with 404,920 new cases estimated in Europe during 2020 (European Union. (2021); European Cancer Information System (ECIS); https://ecis.jrc.ec.europa.eu [accessed 06 December 2022]) and 281,520 new cases in the USA during 2021 (Siegel et al., 2021), where 137,808 breast reconstruction surgeries were performed in 2020 (American Society of Plastic Surgery. (2020); Plastic Surgery Statistic Report; https://www.plasticsurgery.org/documents/News/Statistics/2020/reconstructive-procedure-trends 2020.pdf [accessed 3 January 2023]). Even though major advances have been made in systemic treatments, surgical management is always mandatory. Nowadays, indications of breast-conservative surgery and nipple-sparing mastectomy have greatly expanded, with a cumulative oncological recurrence similar to total mastectomy (Shimo et al., 2016; Sisti et al., 2016; Wong et al., 2019). However, mastectomy remains the most widely performed surgical treatment for breast cancer. The loss of the breast and nipple–areolar complex (NAC) is very distressing for most patients and may lead to psychological stress and body shame (Arroyo and López, 2011). Breast reconstructive procedures using prostheses and pedicled or free flaps are clinically well established with excellent esthetic outcomes. Nevertheless, NAC reconstruction, which is the last and crucial step of the breast reconstruction, is challenging due to a major lack of guidelines (Carlson et al., 2014; Sisti et al., 2016; Kristoffersen et al., 2017; Parks et al., 2021).

Being the main landmark of the breast and a major symbol of femininity, the NAC is defined by the specific tridimensional shape of the nipple, surrounded by the darker pigmented halo of the areola. The skin of the latter is lifted by the delicate reliefs of Montgomery’s tubercles and is highly sensitive. Moreover, major interindividual variations exist in NAC size, projection, and morphology, so this anatomical entity is a significant marker of the breast personal identity. Therefore, NAC reconstruction is essential for women, improving the wellbeing, physical attractivity, and femininity, associated with a positive body image and sexual desire (Wellisch et al., 1987; Didier et al., 2009; Sisti et al., 2016; Satteson et al., 2017), and resulting in higher general and esthetic satisfaction than breast reconstruction alone (Jabor et al., 2002; Sisti et al., 2016; Kristoffersen et al., 2017). Many surgical techniques, using one-or-two-step surgery, have been described for years to reconstruct the NAC (Nimboriboonporn and Chuthapisith, 2014; Sisti, 2020). Most common techniques use tattoos, autologous local skin flaps, or a contralateral nipple graft (Nimboriboonporn and Chuthapisith, 2014; Sisti et al., 2016; Sisti, 2020). Auricular or costal cartilages have been used to enhance the projection of the reconstructed nipple, as well as silicone gel, polytetrafluorethylene, hyaluronic acid, calcium hydroxyapatite, artificial bone, or acellular dermal matrix (ADM) (AlloDerm^©^), after being recovered with a skin flap (Cheng et al., 2007; Garramone and Lam, 2007; Nimboriboonporn and Chuthapisith, 2014; Collins et al., 2016; Bramhall et al., 2017; Sisti, 2020). However, the outcomes of all these techniques often remain sub-optimal and non-lasting and require further correction surgeries (Jabor et al., 2002; Nimboriboonporn and Chuthapisith, 2014; Kristoffersen et al., 2017). The complication rate of nipple reconstruction varies, depending on the technique used, and is estimated to be 46.9% after grafting, 7.9% after local flap, and 5.3% in the case of a flap with autologous or alloplastic graft augmentation (Sisti et al., 2016). Depigmentation, skin flap necrosis, modification, and, mostly, a loss of nipple projection of 40%–75% have been widely reported and are responsible for variable satisfaction levels (Cheng et al., 2007; Garramone and Lam, 2007; Carlson et al., 2014; Nimboriboonporn and Chuthapisith, 2014; Collins et al., 2016; Sisti et al., 2016; Bramhall et al., 2017; Kristoffersen et al., 2017; Komiya et al., 2021).

To avoid these hazards, we hypothesized that tissue engineering techniques, such as the decellularization and recellularization process (DRP) (Badylak et al., 2011; Crapo et al., 2011; Pileggi, 2014), could create a new transplantable 3D NAC allograft, corresponding intrinsically to an ADM. Indeed, the DRP (Crapo et al., 2011) has been described, for several decades, on skin (Dussoyer et al., 2020) and, more recently, on human composite tissues (Duisit et al., 2017; 2018a; Gerli et al., 2018). This process aims to create acellular scaffolds by removing the immunogenic components from native tissues while preserving their extracellular matrix (ECM) architecture and composition. The so-obtained scaffolds can then be seeded with specific autologous cells, thus generating a non-immunogenic living and functional tissue (Badylak et al., 2011). Acellular dermal matrices (e.g., AlloDerm^©^), similarly generated by decellularization, have been clinically used for decades in breast reconstructive surgery without signs of immune rejection and are considered non-toxic and safe for the patients (Wainwright, 1995; Carruthers et al., 2015; Ibrahim et al., 2015; Boháč et al., 2018; Dussoyer et al., 2020; Gierek et al., 2022; Petrie et al., 2022).

Bioengineering techniques were recently applied to rhesus macaque (Pashos et al., 2017; 2020), porcine (Oganesyan et al., 2023), and human (Caronna et al., 2021) NACs, with satisfying outcomes concerning cellular clearance and ECM preservation, as well as in terms of the *in vivo* implantation of the apical nipple part in a narrow skin defect model of 1 cm^2^, which showed re-epithelialization and neo-vascularization by the host tissue after 6 weeks, as in normal wound healing (Rodrigues et al., 2019). However, a human decellularized nipple–areolar complex graft, preserving its specific native molecular characteristics and 3D macro- and microarchitecture, could be an ideal solution to improve the outcomes of current NAC reconstruction after breast cancer. This three-dimensional acellular dermal scaffold could indeed be easily used afterward as a dermal graft (Wainwright, 1995) on the reconstructed breast shape, without the need of a vascular pedicle to support its transplantation (Jank et al., 2017; Pashos et al., 2020; Caronna et al., 2021).

In this work, human NACs were decellularized using sequential detergent agitating baths to generate acellular NAC scaffolds. Cellular clearance, preservation of the ECM architecture, main ECM proteins and growth factors, as well as the residual detergents, were assessed using histological and biochemical methods. *In vivo* biocompatibility and the immunogenic response of the decellularized scaffolds were analyzed after subcutaneous implantation in recipient rats for 30 days and compared with human native NACs. The *in vitro* cytocompatibility of decellularized matrices was assessed by seeding human fibroblasts on their hypodermal side for 7 days, while the *in vitro* regeneration of a new epidermis was studied by seeding human keratinocytes on their epidermal side for 10 days using the reconstructed human epidermis (RHE) technique.

## 2 Materials and methods

### 2.1 Nipple–areolar complex harvesting and decellularization

#### 2.1.1 Human specimens and animal experimentation

Human specimen harvesting: A total of 24 NACs were harvested from 15 cadaveric donors (six women and nine men; mean age: 82.5 years, range 60–100) received at the UCLouvain Human Anatomy Department (IRB00008535, Brussels, Belgium), following the local ethics committee authorization. All deceased donors had provided their consent for their bodies to be used after death and donated for medical research. Animal experimentation: The animal study was conducted following the authorization of the local ethics committee of UCLouvain (ref. 2021/UCL/MD/067, Brussels, Belgium) in accordance with Belgian (Royal Decree, September 2004) and European legislation (Directive-2010–63/UE).

#### 2.1.2 Nipple–areolar complex decellularization

A circular incision was made 0.5–1 cm from the areola border. The NAC was dissected from the hypodermis. Thereafter, excessive fat and connective tissue were removed. Of the 24 human NACs harvested, 15 NACs were decellularized (d-NAC), while 9 NACs were used as native controls (n-NAC) for histology, biochemical assays, and *in vivo* experiments. NACs submitted to the decellularization process were placed in a 250-mL glass jar filled with the following specific solutions. The complete decellularization process was performed on an orbital agitator (200 rpm) at room temperature (RT). The NACs were rinsed in heparinized saline serum (30 UI/L) for 1 h, then washed with deionized water (DIW), and stored at −80°C. Thawing was performed at 37°C under agitation in DIW for 1 h, followed by 1% sodium dodecyl sulfate (SDS) (*27,926.295, VWR*) for 72 h, changed every 12 h, 1% Triton X-100 (M143, VWR) for 24 h, DIW for 24 h, 2-propanol (20922.364, VWR) for 4 h, DIW for 1 h, phosphate-buffered saline (PBS) for 72 h, type I bovine DNAse (11284932001, Roche, Sigma-Aldrich) (25 mg/L in 0.9% NaCl) at 37°C for 4 h, and then finally, 2 h of PBS rinsing at RT. The samples were preserved in PBS at 4°C.

#### 2.1.3 Sterilization of decellularized scaffolds

The scaffolds used for cell culture or *in vivo* implantation were incubated overnight in 0.1% peracetic acid and washed in three baths of sterile DIW, followed by five baths of PBS (59321C, Sigma-Aldrich) containing 100 U/mL of penicillin/streptomycin (P/S) (15140122, Thermo Fisher Scientific) and 2.5 μg/mL of amphotericin B (15290–026, Thermo Fisher Scientific).

### 2.2 Characterization of the decellularized nipple–areolar complex ECM

#### 2.2.1 Tissue sampling

For histology, central strip biopsies of native control (n-NAC) and decellularized (d-NAC) nipple–areolar complexes involving the half nipple and peripheral skin were performed. For DNA, ECM proteins, and residual SDS quantifications, three full-thickness random biopsies were performed per n-NAC and d-NAC and then freeze-dried.

#### 2.2.2 Histology

After fixation in 4% formalin (9713.9010, VWR) for 48 h, the samples were paraffin-embedded, sliced into 5-μm sections, and stained with hematoxylin and eosin (H&E) and Masson’s trichrome (MT). For immunohistochemistry (IHC) and immunofluorescence (IF), after deparaffinization, endogenous peroxidases were inhibited with 3% hydrogen peroxide in methanol. The sections were then exposed to proteinase K antigen retrieval, and aspecific antigen-binding sites were blocked using a solution of 5% BSA (albumin fraction V, 3854.3, Carl Roth) in TBS/Tween 20 (663684B, VWR) at RT for 30 min. The sections were then incubated with anti-MHC class I + HLA A+ HLA B (1:200; Abcam, ab134189, RRID:AB_3073854), anti-collagen I (1:1,500; Abcam, ab138492, RRID:AB_2861258), anti-collagen IV (1:500; Abcam, ab6586, RRID:AB_305584), anti-laminin (1:100; Abcam, ab11575, RRID:AB_298179), anti-fibronectin (1:200; Abcam, ab23751, RRID:AB_447656), anti-CD3 (1:100; Abcam, ab828, RRID:AB_306429), anti-CD31 (1:2,000; Abcam, ab182981, RRID:AB_2920881), anti-CD68 (1:600; Abcam, ab31630, RRID:AB_1141557), and anti-pancytokeratin (1:400; Dako Agilent, M3515, RRID:AB_2132885) primary antibodies at 4°C overnight, followed by incubation with a peroxidase-conjugated anti-rabbit secondary antibody (ready to use, 100 µL per section, EnVision, Dako Agilent, K4003, RRID:AB_2630375) for all the primary antibodies, except the anti-pancytokeratin and anti-CD68 antibodies, which were incubated with an anti-mouse secondary antibody (ready to use, 100 µL per section, EnVision, Dako Agilent, K4001, RRID:AB_2827819) and an anti-mouse horseradish peroxidase (HRP) antibody (1:500; Jackson ImmunoResearch, 715–035–151, RRID:AB_2340771), respectively. They were then revealed using 3,3′-diaminobenzidine (DAB) peroxidase substrate (Dako Angilent, K3468) for IHC or using Alexa Fluor 488/555/647-conjugated tyramide (Invitrogen, Thermo Fisher Scientific*,* B40953, B40955, and B40958) to perform multiplex IF. Nuclei were counterstained with hematoxylin (IHC) or 4′,6-diamidino-2-phenylindole dihydrochloride (DAPI) (1:1,000; Sigma-Aldrich) (IF). The slides were mounted with Entellan New (Merck, 1079610100) or fluorescence mounting medium (Dako Agilent, S302380-2). All H&E, MT, and IHC sections were captured using a slide scanner (SCN400, Leica Biosystems, Germany), and multiplex IF slides were digitized using a fluorescence slide scanner (Axio Scan.Z1, Zeiss, Germany) or visualized using a fluorescence microscope (Axio Imager.Z1, Zeiss, Germany).

#### 2.2.3 DNA quantification

DNA was extracted from 25-mg fresh freeze-dried biopsies using DNeasy^®^ Blood & Tissue Kits (69506, QIAGEN, Italy). The biopsies were incubated with proteinase K solution at 56°C overnight. After the addition of buffer and ethanol, the samples were transferred to a spin column filled with buffers, and repeated elutions were performed. The final amount of the extracted DNA was assessed using a Quant-iT PicoGreen DNA Assay Kit (L3224, Thermo Fisher Scientific) according to the manufacturer’s protocol. Fluorescence was measured at 480 nm/520 nm using a microplate reader (SpectraMax i3, Molecular Devices, United States). Three readings by plate were performed. The results were expressed as the mean DNA amount in ng/mg dry weight ±standard deviation (SD) (n = 11 d-NACs and n = 5 n-NACs).

#### 2.2.4 ECM proteins quantification

The collagen content was quantified from 20-mg fresh freeze-dried biopsies using the QuickZyme Total Collagen assay (QZBTOTCOL2, QuickZyme, Netherlands). The biopsies and standards were hydrolyzed in 6 M HCl at 95°C overnight and then centrifuged. The supernatants were diluted with DIW to obtain a concentration of 4 M. The assay buffer solution was added to each sample. After incubation on an agitation plate at RT for 20 min, the detection reagent was added to each sample and incubated at 60°C for 1 h. The final absorbance was measured at a wavelength of 570 nm using a microplate reader (SpectraMax i3, Molecular Devices, United States). The glycosaminoglycan (GAG) content was quantified from 25 mg fresh freeze-dried biopsies using a Blyscan Sulfated-GAG assay kit (B1000, Biocolor Ltd, Northern Ireland). The samples were digested with papain solution at 65°C overnight. After centrifugation, the supernatants were mixed with Blyscan blue dye reagent, and the samples were incubated on an agitation plate at RT for 30 min and then centrifuged. The pellets were collected, and the dissociation reagent was added to the samples, which were incubated on an agitation plate at RT until complete dissociation. The final absorbance was measured at 630 nm of wavelength. Elastin content was assessed from 10-mg fresh freeze-dried biopsies using a Fast Elastin Assay kit (F2000, Biocolor Ltd., Northern Ireland). The samples were digested twice with 0.25 M oxalic acid at 100°C for 1 h before supernatant collection. The elastin of total extraction was precipitated by adding an equal volume of precipitating reagent to each sample, followed by incubation at RT for 15 min and centrifugation. The dye reagent solution was added to the pellets, incubated on an agitation plate at RT for 90 min, and then centrifuged. The pellets were finally mixed with the dye dissociation reagent and incubated at RT for 10 min. The final absorbance was measured at a wavelength of 510 nm. All kits were used following the manufacturer’s protocols, and for each assay, three readings by plate were performed. The results were expressed as the mean collagen, GAG, or elastin content in µg/mg dry weight ±SD (n = 5 d-NACs and 5 n-NACs).

#### 2.2.5 Human growth factors quantification

Biopsies (50 mg) of fresh decellularized scaffolds (n = 4 different donors, 1 biopsy/d-NAC) and native controls (n = 3 different donors, 1 biopsy/n-NAC) were lysed using a radioimmunoprecipitation assay (RIPA) buffer containing a protease inhibitor cocktail and Pho-Stop at 4°C for 2 h, followed by six cycles of homogenization at 7,200 rpm using a Precellys homogenizer (Bertin Technologies SAS, France). The supernatants were collected, and the total protein concentration of each sample was determined using a Pierce BCA Protein Assay Kit (23227, Thermo Fisher Scientific) following the manufacturer’s protocol. An amount of 60 µg of proteins was processed using Human Growth Factor (GF) Array C1 (AAH-GF-1-2, RayBiotech, United States) according to the manufacturer’s protocols and described methods^34,35^. The assay membranes were blocked with the blocking buffer solution at RT for 30 min before adding 60 µg of proteins to 1 mL of RIPA per membrane, followed by incubation at RT for 2.5 h. Thereafter, the membranes were washed three times in wash buffer I and twice in wash buffer II before being incubated with the biotin-conjugated antibody cocktail at 4°C overnight. The membranes were washed as mentioned above and incubated with HRP–streptavidin at RT for 2 h. After a final wash to remove residual reagents, the membranes were transferred on a plastic sheet, incubated with the Detection Buffer C&D for 2 min, and visualized with enhanced chemiluminescence (ECL) (RPN2109, VWR) on CL-XPosure Films (34091, Pierce). All incubation steps were performed on an agitating plate. The results were calculated following the manufacturer’s protocols, using the previously described methods (Di Meglio et al., 2017; Manon et al., 2023), and using ImageJ software. Each GF has two densitometry spots per assay, and the mean density for each GF per sample was calculated. Each GF mean density was then subtracted from the background density and normalized to the positive control as well as the ratio of the total protein amount to the weight of each sample. The results were expressed as the mean density of each GF for all d-NACs and n-NACs ±SD. Growth factors and cytokines detected by the Human GF Array C1 (AAH-GF-1-2, RayBiotech, United States) were amphiregulin, bFGF, beta-NGF, EGF, EGFR, FGF-4, FGF-6, FGF-7 (KGF), GCSF, GDNF, GM-CSF, HB-EGF, HGF, IGFBP-1, IGFBP-2, IGFBP-3, IGFBP-4, IGFBP-6, IGF-1, IGF-1 R, IGF-2, M-CSF, M-CSF R, NT-3, NT-4, PDGFR-alpha, PDGFR-beta, PDGF-AA, PDGF-AB, PDGF-BB, PLGF, SCF, SCF R, TGF-alpha, TGF-beta 1, TGF-beta 2, TGF-beta 3, VEGF-A, VEGFR2, VEGFR3, and VEGF-D.

#### 2.2.6 Residual SDS quantification

SDS is a strong cytotoxic detergent and needs to be washed from the acellular matrix at the end of the decellularization process in order to not be deleterious during the recellularization steps or after *in vivo* implantation. The residual SDS in the decellularized scaffolds was quantified using the methylene blue active substances (MBAS) assay according to previously published protocols (Andrée et al., 2014; Rougier et al., 2023). Fresh biopsies (n = 3 different d-NACs) of 40 mg were carried out, freeze-dried, and then, digested in 1 mL of proteinase K (10 μL proteinase K 19.1 mg/mL in 30 mM Tris, pH 8.0) (1.07393.0010, Merck, Sigma-Aldrich) at 50°C overnight. A standard curve was set up to extrapolate the SDS amount: 1 μL of SDS 0.5%, 0.25%, 0.125%, 0.0625%, 0.0313%, 0.01565%, 0.0078%, 0.0039%, and 0% (DIW) was mixed with 249 μL of DIW. All standards were further processed like the samples. Then, 250 μL of samples or standards were mixed with 250 μL of methylene blue and vortexed. Thereafter, 500 μL of chloroform (1.02445, VWR) was added to each sample or standard and vortexed. Finally, 200 μL of the chloroform layer were placed in a 96-well plate, and the absorbance of each samples was measured at 651 nm using a microplate reader (SpectraMax i3, Molecular Devices, United States). Residual SDS in the ECM was calculated from the standard curve and expressed as the mean residual SDS in μg/mg dry weight ±SD and in μg/mL ± SD for the SDS concentration in the digested proteinase K solution (n = 3).

### 2.3 Biocompatibility of decellularized nipple–areolar complexes

#### 2.3.1 *In vivo* subcutaneous implantation of decellularized NACs

The *in vivo* biocompatibility of the bioengineered acellular scaffolds was evaluated after the subcutaneous implantation of native and decellularized human NAC patches in Wistar rats. Ten rats were divided into two groups of five rats. After general anesthesia induced by continuous isoflurane ventilation, a median incision was performed following the spine of the rat. A subcutaneous chamber was created. Then, one patch of 8 mm × 8 mm × 5 mm from the native or decellularized human NAC (n = 5 for d-NACs and n = 5 for n-NACs, from five different donors) was implanted per rat. The skin was closed with Donati sutures using 3/0 Vicryl. On post-operative day 30 (POD30), all rats were euthanized by exsanguination under general anesthesia induced by isoflurane gas. Human native and decellularized implants were explanted with their surrounding tissues, fixed in 4% formaldehyde for 48 h, and stained for H&E, CD68, CD3, CD31, and DAPI (Histology). The samples on each histological slide were manually delineated while taking care to remove artifacts from the analysis. CD68- and CD3-stained positive cells, corresponding to pan-macrophages and pan-lymphocytes, respectively, were quantified using QuPath software (v0.3.0., University of Edinburgh) (Bankhead et al., 2017) on each delineated tissue. The results were expressed as the mean number of cells stained for CD3 or CD68 among all cells stained with DAPI per mm^2^. To detect the presence of rat anti-human IgG in the serum, 500 μL of blood was taken by tail puncture on the day of implantation (POD0) and excision (POD30). The collected blood samples were centrifuged at 3,000 rpm for 15 min, and the sera were preserved at −80°C until further use.

#### 2.3.2 Detection of anti-donor antibodies (IgG) by flow cytometry

The presence of rat anti-human IgG was evaluated in POD0 and POD30 sera by flow cytometry, as previously described (Duisit et al., 2018b). Human peripheral blood mononuclear cells (PBMCs), freshly isolated from a healthy donor, were incubated with the recipient serum at RT for 30 min. Before incubation, the serum was decomplemented at 56°C for 35 min. After washing with a fluorescence-activated cell sorting buffer (PBS containing 3.5% fetal bovine serum (FBS) (10270–106, Thermo Fisher Scientific) and 1% sodium azide), saturating amounts of Alexa Fluor 488 goat anti-rat IgG (H + L) and Cross-Adsorbed Secondary Antibody Alexa Fluor 488 (A-11006, Thermo Fisher Scientific) were added and incubated at RT for 30 min and then washed twice. Each analysis included the appropriate Alexa Fluor 488-conjugated antibody with only PBMCs, for non-specific reactions. Cells were isolated and analyzed with BD FACSCalibur (BD Biosciences Benelux NV, Belgium) driven by CellQuest Pro software (BD Biosciences Benelux NV, Belgium). A positive reaction was defined as a shift of more than 10 channels in mean fluorescence intensity when testing donor lymphocytes with post-transplantation (POD30) serum and comparing with pre-transplant serum (POD0).

### 2.4 Recellularization of decellularized nipple–areolar complexes

#### 2.4.1 Cell culture

Cryopreserved human keratinocytes (HEKa) (C0055C, Thermo Fisher Scientific) and human fibroblasts (HFs), isolated by abdominoplasty and graciously provided by Pr. Poumay (UNamur, Belgium), were thawed at 37°C. HEKa were mixed with cold keratinocyte medium (KCM) corresponding to EpiLife medium (MEPI500CA, Thermo Fisher Scientific) containing 0.06 mM CaCl_2_, HKGS (S0015, Thermo Fisher Scientific), and 1% P/S. HFs were mixed in a fibroblast culture medium (FCM) consisting of DMEM (BE12-604F, Lonza, Westburg, Netherlands) containing 10% FBS (10270–106, Thermo Fisher Scientific), 1% L-glutamine (BE17-605E, Lonza, Westburg, Netherlands), and 1% P/S. The cells were cultured in a cell culture (37°C, 5% CO_2_) incubator, and the medium was changed every 2 days.

#### 2.4.2 *In vitro* cytocompatibility of decellularized NACs

Sterile 1-cm^2^ acellular ECM discs were incubated in the FCM overnight. An amount of 5 × 10^5^ HFs suspended in 1,000 µL of FCM were seeded on the hypodermal side of the scaffolds (n = 5) placed in a 48-well culture plate or directly in the culture well used as the control (n = 5). The next day, the ECM discs were transferred to a 12-well culture plate and cultured for 7 days. They were analyzed through live/dead staining (L-3224, Life Technologies, Thermo Fisher Scientific) according to the manufacturer’s protocol, assessed using a fluorescence microscope (Axio Imager.Z1, Zeiss, Germany), fixed in 4% formaldehyde, and stained with H&E. The cell viability of each seeded ECM disc and control well was evaluated on day 7 on four different live/dead acquisitions per sample, taken at five-fold magnification as previously described (Rougier et al., 2023). They were then quantified using FIJI^®^ software. The cell viability (%) corresponded to the ratio of the green area (living cells) to the sum of the green and red (dead cells) areas (total number of cells), after removing the artifacts. The results were expressed as the mean percentage (%) of cell viability of the four live/dead acquisitions ±SD for all seeded ECMs and control wells. Cell proliferation was assessed by a PrestoBlue Assay (A13262, Thermo Fisher Scientific) on days 3, 5, and 7 by seeding 2 × 10^5^ HFs on ECM discs (n = 3) or in culture wells (n = 3) as the control. The FCM was removed and replaced by 200 µL of PrestoBlue solution (1:10 FCM) and incubated for 1.5 h. A measure of 100 μL of the supernatant was transferred to a 96-well opaque plate, and the fluorescence intensity was read at 560/590 nm using a microplate reader (SpectraMax i3, Molecular Devices, United States). The results were expressed as the mean fluorescence intensity ±SD.

#### 2.4.3 *In vitro* epidermis regeneration of decellularized NACs

New epidermis formation was investigated by applying the RHE technique (De Vuyst et al., 2013). Sterile 1-cm^2^ acellular ECM discs (n = 5) were incubated in KCM overnight and then placed in a 48-well culture plate. The dermis was seeded with 1 × 10^6^ HEKa suspended in 1,000 µL of medium, which was changed every day. After 3 days, the seeded ECMs were transferred to a 12-well culture plate. EpiLife medium containing HKGS, 10 ng/mL of KGF, 1.5 mM calcium, 91.4 μg/mL vitamin C, and 1% P/S was added until the epidermis level was reached, creating an air–liquid interphase. After 7 days of culture, the samples were fixed and stained with H&E or subjected to anti-pancytokeratin IF staining (see Section 2.2.2).

### 2.5 Statistics

All statistical analyses were performed using GraphPad Prism 8 (GraphPad software, United States). Data are presented as the mean ± SD. Normality was verified using the Shapiro–Wilk test, with specific unpaired t-tests applied thereafter. For all tests, statistical significance was *p* < 0.05.

## 3 Results

### 3.1 Characterization of the decellularized nipple–areolar complex

#### 3.1.1 Macroscopic aspect

**FIGURE 1:**
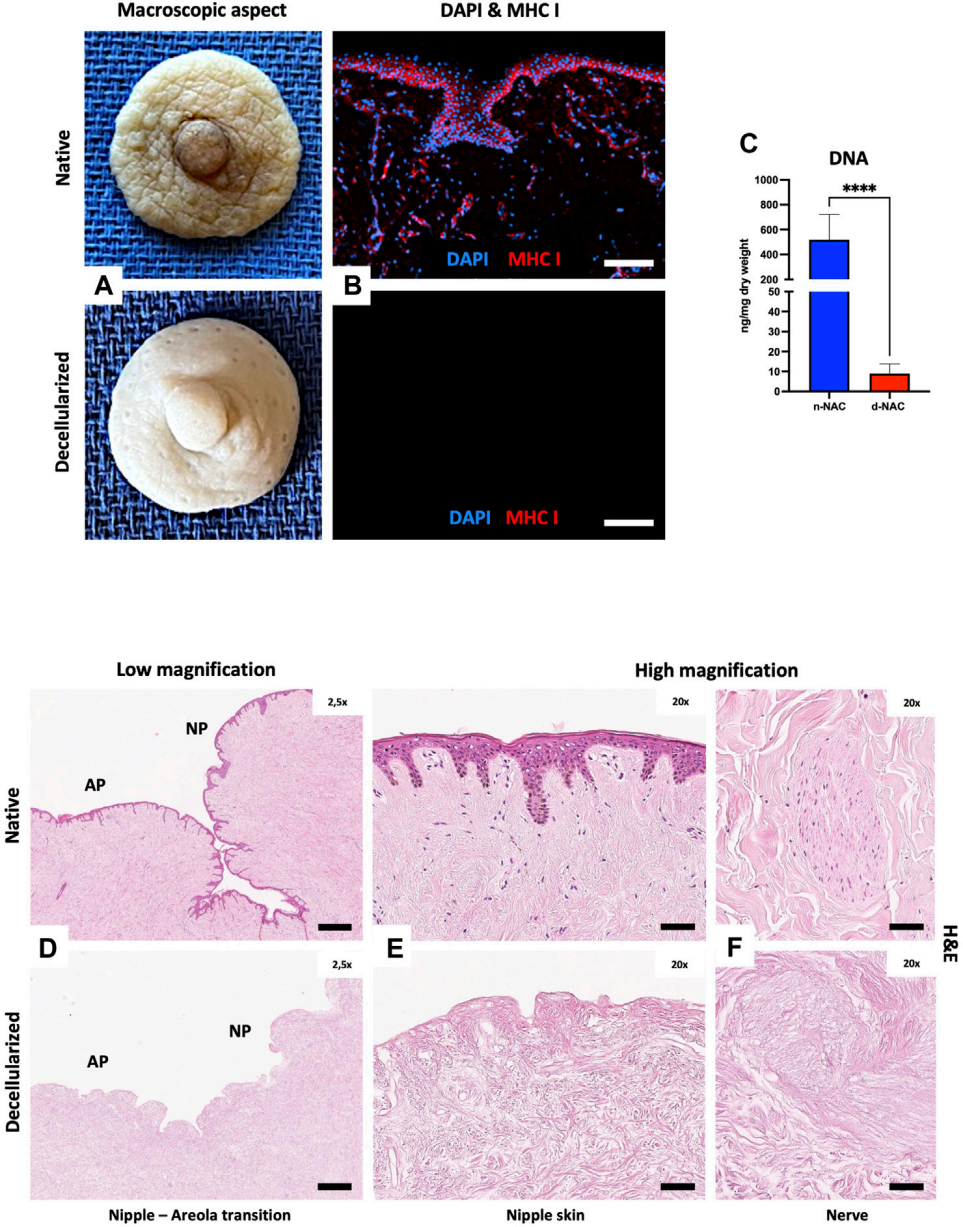
Human NAC decellularization and cell clearance. **(A)** Macroscopic aspect of the harvested native (top) and decellularized (bottom) NAC, which appears white and is entirely de-epithelialized. **(B)** DAPI (blue) and MHC class I + HLA A+ HLA B (red) immunofluorescence staining confirms the total decellularization by the absence of nuclei and MHC-I + HLA A + HLA B antigens in decellularized tissues (bottom) compared to the native tissues (top) (scale bar = 100 μm). **(C)** DNA quantification in native NAC (n-NAC) (n = 5) versus decellularized NAC (d-NAC) (n = 11) scaffolds shows a significant DNA reduction (98%) after decellularization. The DNA concentration is expressed in ng/mg dry weight. Error bars: SD; ****p < 0.001. **(D–F)** H&E-stained sections of n-NAC (top) and d-NAC (bottom) at low magnification **(D)** with the areolar part (AP) and nipple part (NP) of the NAC. High magnification of H&E-stained sections focused on the epidermis **(E)** and nervous ramifications **(F)** of native (top) and decellularized (bottom) NACs. Both magnifications confirm the complete decellularization (scale bar for D = 400 μm and for E–F = 50 μm).

After decellularization, nipple–areolar complex grafts appeared completely white and entirely de-epidermalized. Residual lobular fat tissue was also entirely removed, and the dermal and hypodermal sides appeared white. The nipple and Montgomery’s tubercles, responsible for the specific human NAC 3D morphology, were fully preserved (Figure 1A).

#### 3.1.2 Cellular clearance

Decellularization was highlighted by the absence of nuclear staining and negative MHC class I + HLA A+ HLA B IF staining in acellular scaffolds compared to native tissues (Figure 1B). Effective decellularization was confirmed by a major and significant decrease of 98.3% in DNA amount (*p* < 0.0001) in decellularized scaffolds compared to native tissues. Indeed, the DNA amount decreased from 518.3 ± 204.9 ng/mg dry weight in control native grafts to 9.37 ± 5.14 ng/mg dry weight in acellular scaffolds (Figure 1C). H&E-stained sections at low and high magnifications also showed complete cell removal, with the absence of nuclear staining in both nipple and areolar parts (Figures 1D, E). Residual acellular nervous structures were also visible in decellularized scaffolds, compared to the native tissue (Figure 1F).

#### 3.1.3 ECM preservation

**FIGURE 2:**
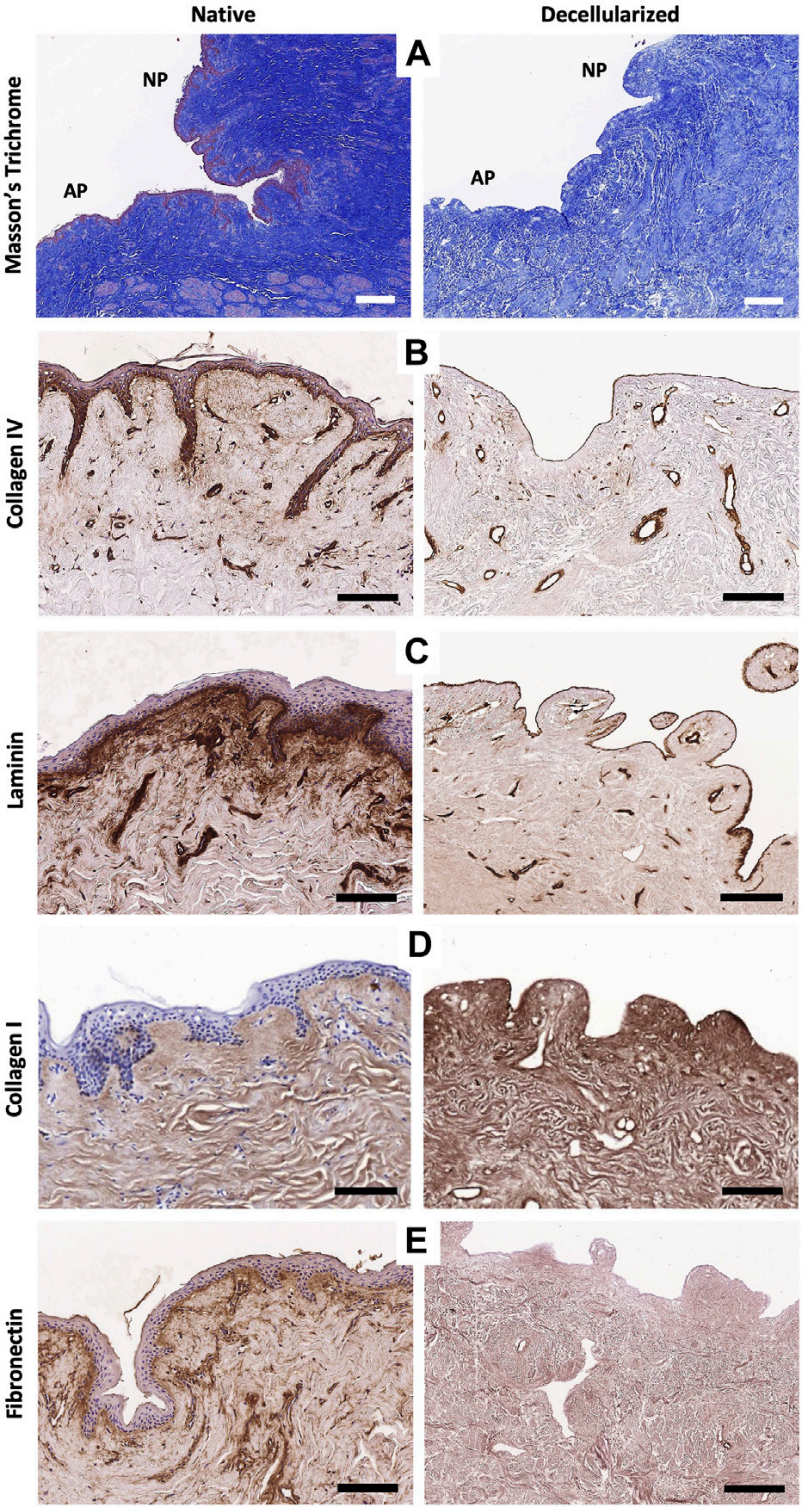
Extracellular matrix microarchitecture preservation. **(A)** Masson’s trichrome staining of native (left) and decellularized (right) NACs at low magnification, with the AP and NP, shows the preservation of the NAC microarchitecture and collagen fibers in decellularized tissues (scale bar = 100 μm). **(B–E)** Immunohistochemistry stainings of the main ECM proteins evaluating the preservation of type IV collagen **(B)**, laminin **(C)**, type I collagen **(D)**, and fibronectin **(E)** in native tissues (left) compared with decellularized tissues (right) (scale bar = 200 μm).

**FIGURE 3:**
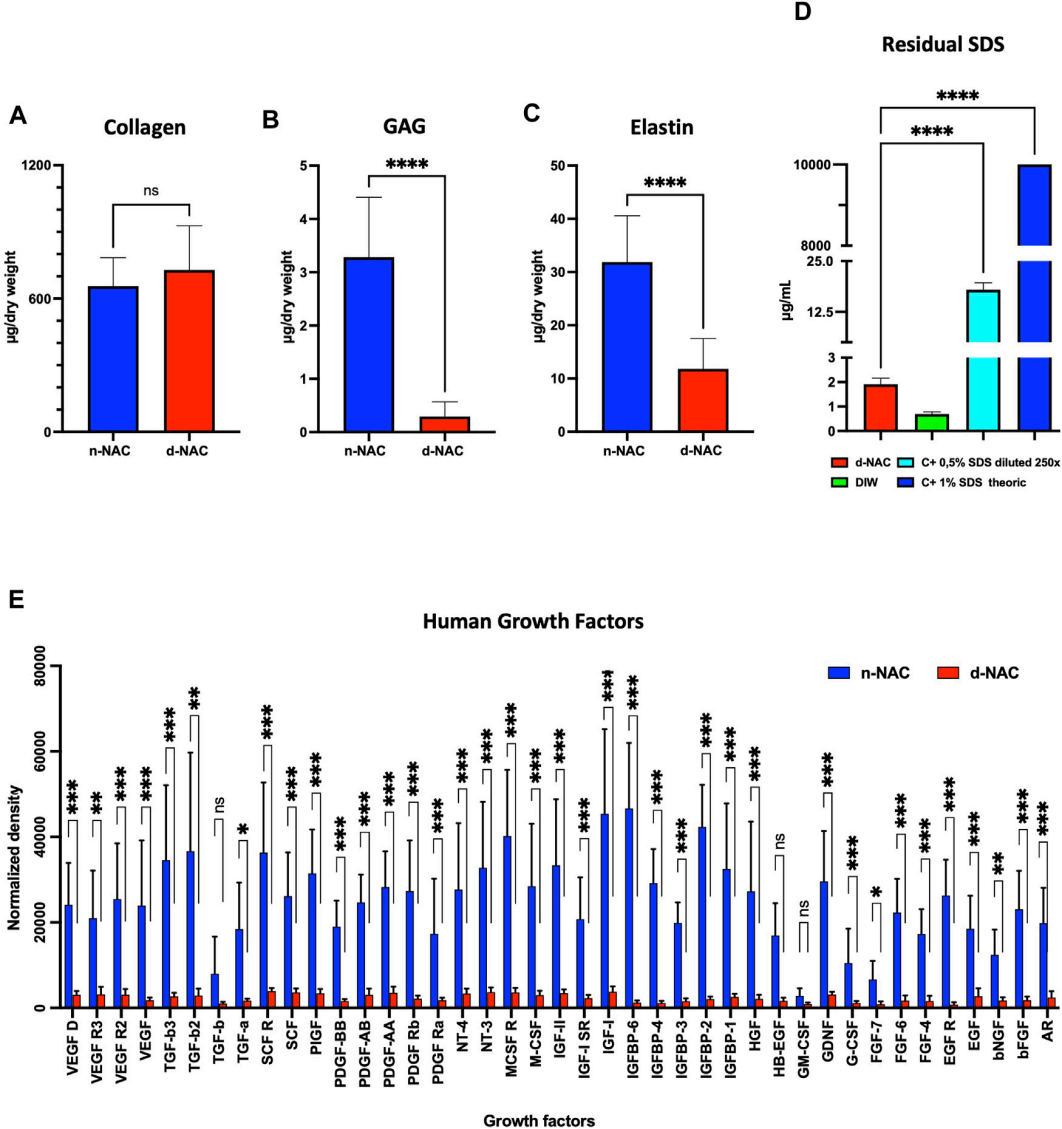
Extracellular matrix component preservation. **(A–C)** Main ECM protein quantification in n-NACs and d-NACs. **(A)** Collagen quantification shows the preservation of collagen in d-NACs. **(B)** GAG and **(C)** elastin quantifications highlight a significant reduction in decellularized scaffolds compared to the native tissue. Collagen, GAG, and elastin concentrations are expressed in μg/mg dry weight. Error bars: SD; ****p < 0.001, ns = not significant. **(D)** Residual SDS quantification in d-NAC shows a significantly very low amount of SDS residues in acellular scaffolds after the last washing step of the decellularization protocol, confirming the non-toxicity of the decellularized scaffolds. Residual SDS is expressed in μg/mg dry weight. Error bar: SD; ****p < 0.001, n = 3. **(E)** Quantification of human growth factors: All 41 human GFs were detected before (n = 3) and after decellularization (n = 4), with a significant reduction in 38/41 GFs and an insignificant reduction in 3/41 GFs. The results are expressed as the mean density. Error bar: SD; ****p < 0.0001, ***p < 0.001, **p < 0.01, *p < 0.05, and ns = not significant.

MT-stained sections of decellularized NACs also highlighted cellular removal but mostly showed the preservation of their microscopic architecture, including collagen fibers, nerves, glands, and vessels (Figure 2A). Type IV collagen and laminin immunostaining qualitatively attested the preservation of basal membranes in the dermis, vessel walls, and nerves after decellularization (Figures 2B, C). Type I collagen fibers highlighted by immunostaining were well retained, as shown in the MT-stained sections, while fibronectin staining was decreased after decellularization on immunostained sections (Figures 2D, E). The main ECM proteins (collagen, GAGs, and elastin) were preserved, each with a distinct level of preservation. The collagen content was well preserved after decellularization compared to controls, corresponding to a total collagen content of 655.7 ± 127.9 μg/mg dry weight in control native NACs and 728.7 ± 199.1 μg/mg dry weight in decellularized NACs (*p* = 0.242) (Figure 3A). This fact can be explained by the loss of the cellular compartment and other ECM components. This results in a higher relative amount of collagen in a decellularized tissue than in a native one (Duisit et al., 2017; 2018a). GAGs and elastin content were significantly decreased after decellularization by 90% (*p* < 0.0001) and 63% (*p* < 0.0001), respectively. GAG content decreased from 3.282 ± 1.126 μg/mg dry weight in n-NACs to 0.293 ± 0.273 μg/mg dry weight in d-NACs (Figure 3B), while elastin content decreased from 31.88 ± 8.703 μg/mg dry weight in n-NACs to 11.82 ± 5.70 μg/mg dry weight in d-NACs (Figure 3C).

#### 3.1.4 Residual SDS in acellular scaffolds

The MBAS confirmed the very low concentration of residual SDS, which was measured at 1.912 ± 0.245 μg/mL in the digested matrix, corresponding to an SDS amount of 0.261 ± 0.038 μg/mg dry weight. The residual SDS concentration of the theoretical 1% SDS solution (*p* < 0.0001) used for decellularization was 0.0026% (Figure 3D). Based on this observation, SDS-decellularized matrices can be efficiently washed out with DIW and PBS.

#### 3.1.5 Preservation of the human growth factors

Protein quantification revealed a preservation of 8.79% ± 1.80% of total proteins after decellularization. In addition, this decellularization protocol allowed us to retain the human native growth factors and cytokines in the acellular ECM. Indeed, all the 41 GFs and cytokines analyzed by the assay were detected in decellularized scaffolds, despite the fact that a loss was measured compared with control native tissues: 38 GFs and cytokines were significantly decreased (*p* < 0.05) after decellularization compared to the native controls, while the amount of GM-CSF, HB-EGF, and TGF-B1 was similar to that in native controls (Figure 3E).

### 3.2 Immunocompatibility of decellularized nipple–areolar complex

**FIGURE 4:**
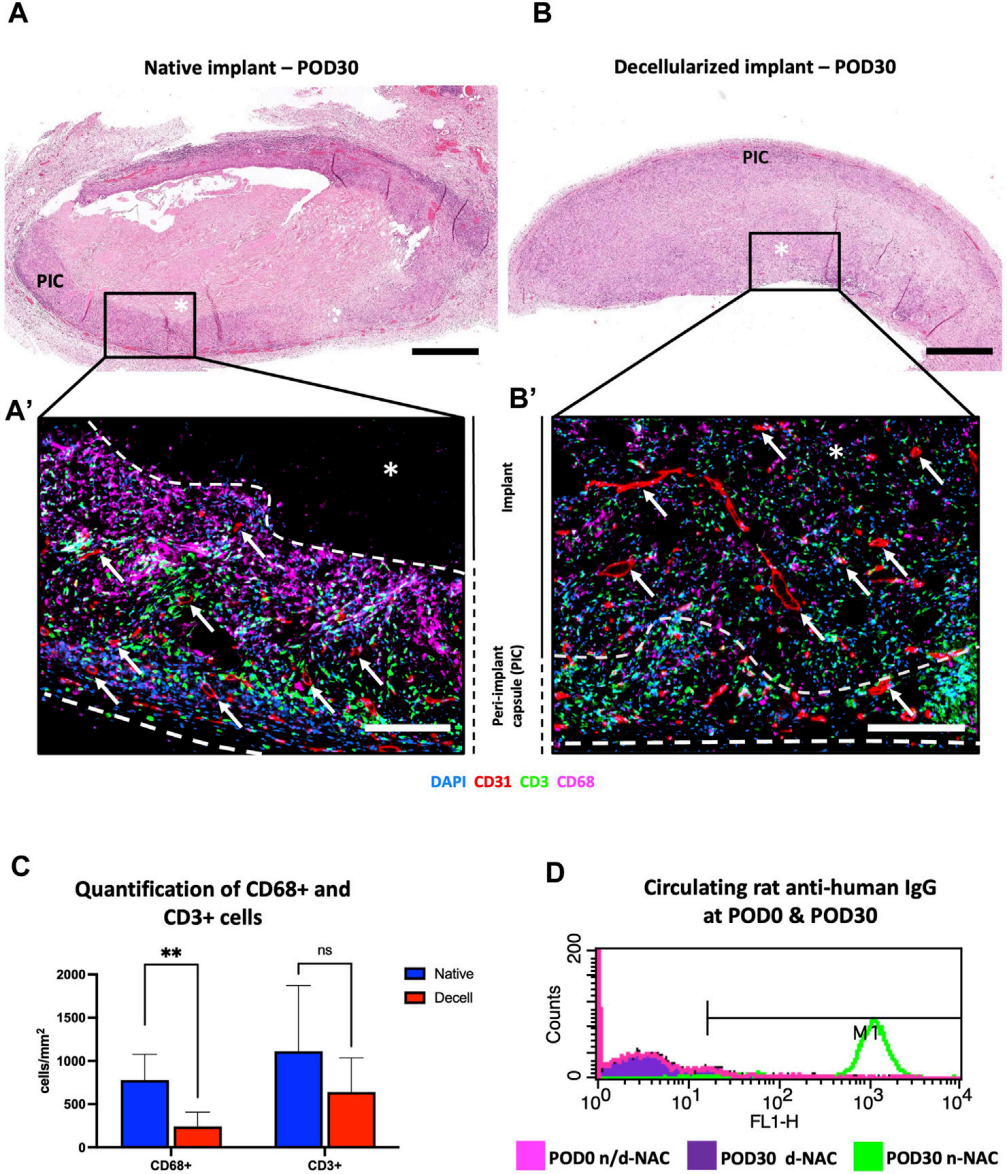
*In vivo* biocompatibility of decellularized NACs subcutaneously implanted in rats. **(A)** H&E-stained native NAC implant on POD30, surrounded by a thick vascularized purple circumferential PIC composed of immune cells and fibrosis with no implant infiltration. * = implant; scale bar = 400 μm. **(B)** H&E-stained decellularized NAC implant on POD30, with complete cell infiltration associated with a thin peripheral vascularized cell layer. * = implant; scale bar = 400 μm. **(A’, B’)** Multiplex immunofluorescence for CD31 (red), CD68 (purple), and CD3 (green) cells in n-NAC **(A’)** and d-NAC **(B’),** showing the formation of a thick peripheral immune cell layer without the penetration of the native implant compared to the complete infiltration of the decellularized scaffold by host cells. Moreover, vessels (CD31^+^, red staining) were found in the n-NAC peripheral layer without the penetration of the implants while they were around and infiltrating the entire thickness of the decellularized scaffolds after 30 days of implantation (scale bars = 50 μm, * = implant, dotted lines = delimitation of the implant, and white arrow = neo-vessels). **(C)** Quantification of positive CD68- and CD3-stained cells in n-NACs (blue) and d-NACs (red) on multiplex immunofluorescence on POD30. The results are expressed in the amount of positive stained cells (CD3^+^ or CD68^+^) per mm^2^. Error bars: SD; **p < 0.001; ns = not significant. **(D)** Flow cytometry of circulating rat anti-human IgG on POD30 showing total immunization after the implantation of native scaffolds (5/5) and the absence of IgG after the implantation of decellularized scaffolds (4/5). Image of flow cytometry is a summary picture of the assay: POD0, both tissues (rose); POD30, n-NACs (green); and POD30, d-NACs (purple).

On POD30, the implants of both groups appeared macroscopically integrated into the neighboring tissue, with a peripheral colonization by thin neo-vessels and a low-inflammatory reaction around the decellularized implants, while control human native implants were encapsulated by a fibrous capsule. Microscopically, the control implants showed an important foreign body reaction. At the periphery of the native implants, numerous immune cells were found in a thick and richly vascularized fibrosis peri-implant capsule (PIC) without penetrating the implant (Figures 4A, A’). In comparison, decellularized implants were diffusely and fully infiltrated by immune and host cells, associated with a thinner peripheral cell layer (Figures 4B, B’). Moreover, a neo-vascularization and micro-vascular colonization with the formation of capillaries, stained for CD31, were found at the periphery and throughout the entire thickness of the decellularized implants (Figure 4B’). This observation confirmed their full revascularization by the recipient after 30 days, compared to the control implants where the neo-vessels were only found in their surrounding capsular layer (Figure 4A’). Quantitatively, immune cells (CD68 cells + CD3 cells) represented 33.39% ± 5.93% and 25.272% ± 7.28% of the total infiltrating cells, respectively, in human native controls and decellularized scaffolds. Additionally, we evidenced a significantly higher infiltration of positive CD68-expressing cells (pan-macrophages) in n-NACs than in d-NACs (779.7 ± 295.9 cells/mm^2^ vs. 276.8 ± 134.8 cells/mm^2^, *p* = 0.0086) and a statistically non-significant difference for positive CD3-expressing cells (pan-lymphocytes) in control human NACs compared to decellularized scaffolds (1,112 ± 760.6 cells/mm^2^ vs. 727 ± 324.7 cells/mm^2^, *p* = 0.328, respectively) (Figure 4C). Nevertheless, high levels of circulating rat anti-human IgG were detected in all specimens of the group implanted with the human native tissues (5/5). Conversely, a low level of IgG was detected in only one specimen of the group implanted with the decellularized scaffolds (1/5) (Figure 4D).

### 3.3 Recellularization of decellularized nipple–areolar complex

#### 3.3.1 *In vitro* biocompatibility of decellularized NAC

**FIGURE 5:**
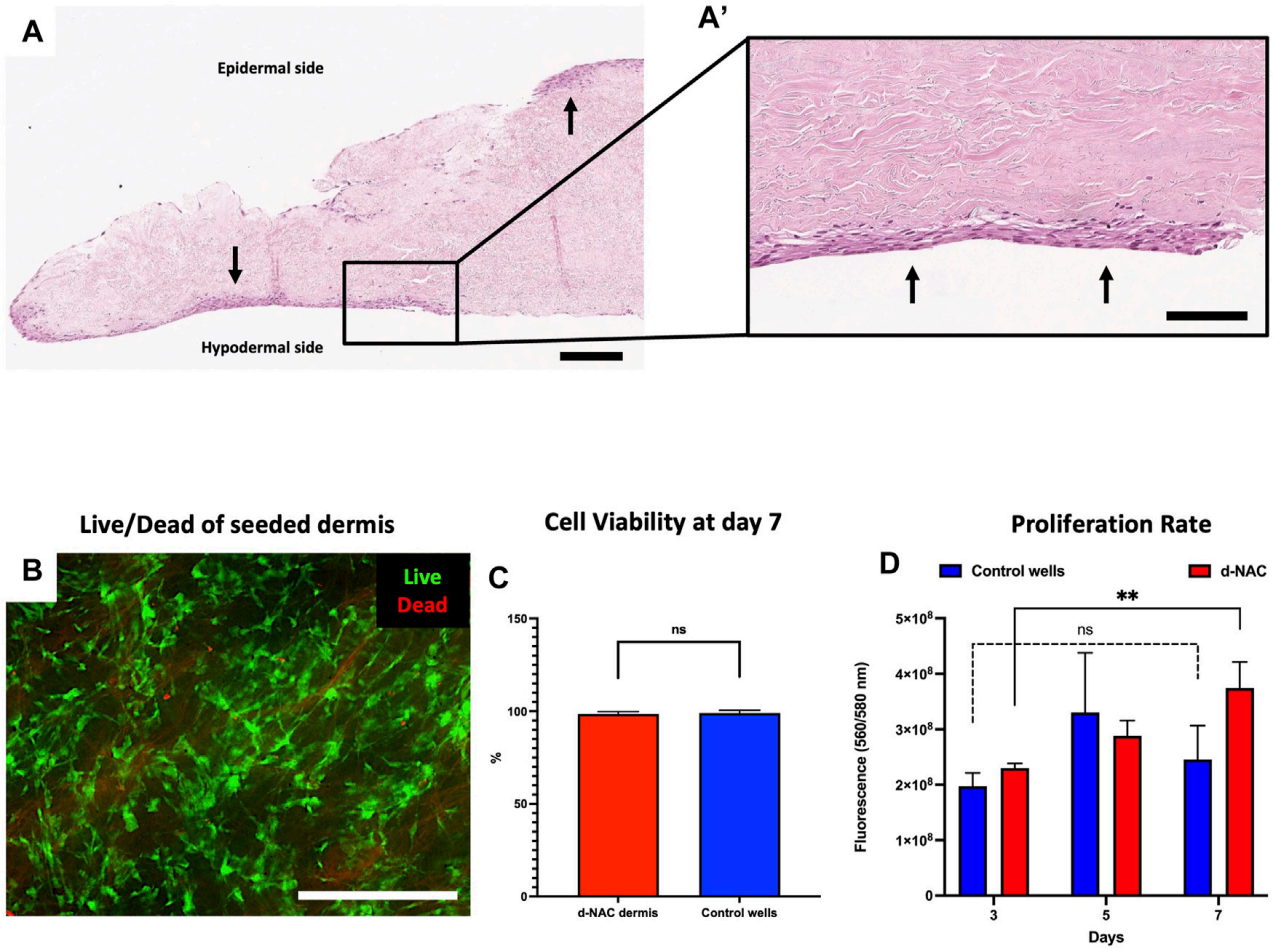
Human fibroblast seeding on the hypodermal side of d-NACs. **(A)** H&E staining of adherent human fibroblasts (arrows) on the acellular hypodermal side and, in some locations, adherent to the epidermal side of d-NACs after 7 days of static culture (scale bar = 200 μm). **(A’)** Higher magnification of the H&E-stained section highlighting adherent fibroblasts forming several cell layers on the hypodermal side of the scaffold (scale bar = 100 μm). **(B)** Live/dead staining of seeded fibroblasts shows a high viability on day 7 of the culture on the scaffold (living cells = green and dead cells = red) (scale bar = 500 μm). **(C)** Cell viability of the seeded dermis: ECM- red and control wells- blue. The results are expressed as the mean cell viability. Error bars: SD; ns = not significant. **(D)** A PrestoBlue cell viability assay realized on seeded d-NACs (red, n = 3) and control culture wells (blue, n = 3) attests the biocompatibility of the produced scaffolds by the increase in metabolic activity during the 7 days of culture. The results are expressed as the mean fluorescence intensity. Error bars: SD; **p < 0.01; ns = not significant.

After 7 days of culture, H&E staining assessed the engraftment and spreading of HFs on the acellular hypodermal side, forming several cellular layers at some locations. Adherent cells were also found on the epidermal side due to their sliding during the seeding (Figures 5A, A’). Live/dead staining showed on the hypodermal side of the scaffold a majority of living cells compared to dead cells (Figure 5B). No significant difference of viability was observed between ECMs and control wells (98.54% ± 1.23% vs. 99.09% ± 1.47%, *p* = 0.0890, respectively) (Figure 5C). The *in vitro* cytocompatibility and the cell proliferation were also confirmed by a PrestoBlue assay. Between days 3 and 7, we observed, in both groups, an increase in fluorescence intensity corresponding to an increase in cell proliferation and, thus, cell amount. The latter were significant in the ECM-seeded group (day 3: 2.3 × 10^8^ ± 8.2 × 10^6^ vs. day 7: 3.7 × 10^8^ ± 4.7 × 10^7^, *p* = 0.0065, expressed as the mean fluorescence intensity ±SD) and insignificant in the control group (day 3: 1.9 × 10^8^ ± 2.4 × 10^7^ vs. day 7: 2.4 × 10^8^ ± 6.1 × 10^7^, *p* = 0.4000, expressed as the mean fluorescence intensity ±SD) (Figure 5D), but no difference was observed between both groups on day 7 (*p* = 0.27). Additionally, after 7 days of culture, we observed significantly increasing proliferation kinetics of 1.63 ± 0.26-fold (*p* < 0.001) in the seeded ECM disc group compared to day 3, while no difference was detected in the control group (1.242 ± 0.22-fold) (*p* = 0.1223). These findings confirmed the *in vitro* biocompatibility of the decellularized scaffolds, highlighted by their non-cytotoxicity and cytocompatibility.

#### 3.3.2 Epidermis recellularization of decellularized NAC

**FIGURE 6:**
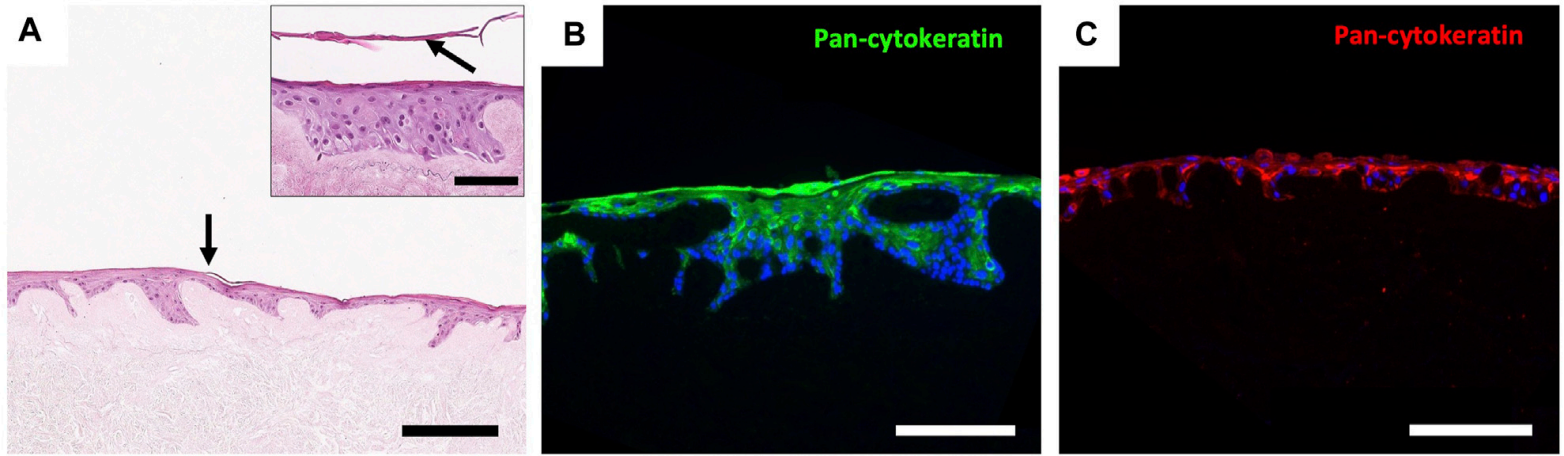
Regeneration of an epidermis using the RHE technique. **(A)** H&E staining of human keratinocytes seeded on the epidermal side of a d-NAC scaffold shows the formation of a stratified epithelium with a superficial squamous layer (arrow) using the RHE technique (scale bar = 200 μm). (**(A)**, right insert) Higher magnification of the regenerated epidermis on d-NAC after 3 days of culture in the medium and then lifted onto an air–liquid interface for 7 days (arrow = desquamating layer) (scale bar = 100 μm). **(B, C)** Pancytokeratin immunofluorescence of the regenerated epidermis on different samples of discs from d-NACs (B in green and C in red), showing different thicknesses of RHE and confirming the expression of cutaneous keratin markers by seeded keratinocytes (scale bar = 200 μm).

After 10 days of culture, H&E staining highlighted the formation of a keratinized stratified squamous epithelium, presenting several layers of keratinocytes and a similar layering as the native epidermis (Figure 6A). Moreover, the reconstructed human epidermis showed positive staining for pancytokeratin (Figures 6B, C), demonstrating the ability of the decellularized scaffold to support the *in vitro* regeneration of a cutaneous epithelium.

## 4 Discussion

In the reconstructive process after breast cancer, nipple–areolar complex restoration is the last challenging step of the procedure, usually considered “*the cherry on the cake,*” but unfortunately delivering less reliable and durable results. In order to improve the current reconstruction techniques, we propose a potential easy solution using a bioengineered scaffold, with composition and tridimensional characteristics similar to those of the native complex, which could be easily generated and implanted as a clinical ADM. In addition to the preliminary data on NAC bioengineering previously collected in animal models (Pashos et al., 2017; 2020), the present study, which is the second study performed on human NACs (Caronna et al., 2021), demonstrates the technical feasibility to decellularize the human NAC while preserving its specific 3D macroscopic morphology, which is critical in terms of esthetic reconstruction. The so-obtained scaffold underwent an original differential cellular seeding on both its hypodermal and epidermal sides. The latter was achieved with human keratinocytes through the *in vitro* RHE technique (De Vuyst et al., 2013) and brings, in a future preclinical perspective, the promise of a lower contraction of the bioengineered NAC after implantation than when secondarily covered by a skin graft or after re-colonization by the host skin, as previously suggested (Pashos et al., 2020; Caronna et al., 2021; Oganesyan et al., 2023).

To generate acellular NAC scaffolds, we used our previously described perfusion decellularization protocols (Duisit et al., 2017; 2018a; 2018b) including 1% SDS, 1% Triton X-100, and DNAse solutions, modified for an agitating bath protocol. Decellularization was confirmed by complete cellular clearance and loss of immunogenic staining, as well as a DNA reduction of 98.3%, which corresponds to a final DNA amount of 9.37 ± 5.14 ng/mg dry weight after decellularization. This DNA amount is under the threshold of 50 ng/mg dry weight established by Crapo et al. (2011), allowing us to consider these scaffolds immunologically safe for transplantation. As described in the literature, a higher amount of residual DNA in decellularized tissues seems to promote inflammation and adverse immune responses (Badylak et al., 2011; Crapo et al., 2011). However, most clinical ADMs used in reconstructive surgeries for decades or even a decellularized porcine skin flap did not seem to induce an immune response (Wainwright, 1995; Eppley, 2000; Moore et al., 2015; Jank et al., 2017; Pashos et al., 2017; Gierek et al., 2022) despite higher DNA amounts than this threshold. Contrariwise, Matracell (Moore et al., 2015) or most experimental bioengineered composite tissue grafts and animal decellularized NACs (Duisit et al., 2017; 2018a; 2018b; Pashos et al., 2017) are below this critical DNA threshold.

The decellularization process is a balance between the most efficient cellular and immunogenic clearance and the best preservation of the ECM. Each of the ECM components indeed plays a key role in promoting the adherence, proliferation, migration, and differentiation of the host or seeded cells (Frantz et al., 2010; Peloso et al., 2016; Karamanos et al., 2021). Furthermore, it favors the integration, revascularization, and re-epithelialization of the decellularized scaffolds after *in vivo* implantation. Regarding the ECM, we noticed an excellent 3D macroscopic preservation of the specific human nipple and areola structure, explained by the retention of the ECM microarchitecture and collagen fiber arrangement, which seems essential to maintain its natural 3D shape. Despite the lower expression of fibronectin after decellularization, we observed complete epidermolysis during the SDS step, compared to other studies on animal NAC bioengineering (Pashos et al., 2017; Caronna et al., 2021) which did not include this step. The epidermolysis allows us to expose the dermis basal membrane while preserving type IV collagen and laminin, which are important for the attachment of cells and keratinocytes and for their proliferation (Vig et al., 2017; Rodrigues et al., 2019; Karamanos et al., 2021).

Using strong detergents, we observed in the decellularized scaffold an insignificant increase of 11% in collagen content (ns) after decellularization, while we observed a significant decrease in GAG and elastin content of 90% (*p* < 0.0001) and 63% (*p* < 0.0001), respectively. These observations regarding the main ECM proteins are also reported in other bioengineered skin tissues (Reing et al., 2010; 2010; Crapo et al., 2011; Jank et al., 2017; Duisit et al., 2018a; 2018b), although not observed by other teams regarding the GAG content (Bondioli et al., 2014; Pashos et al., 2017; Belviso et al., 2020). In comparison, macaque decellularized NACs (Pashos et al., 2017) were generated using sodium deoxycholate (SDC), Triton X-100, and DNAse solutions, while porcine NACs (Oganesyan et al., 2023) were decellularized using hypertonic NaCl, various sequential SDS concentrations, EDTA, Triton X-100, and DNAse. In these works, the collagen content was preserved at 128% (ns) and significantly decreased by 33% (*p* < 0.001), the elastin content was significantly reduced by 69% (*p* < 0.001) and 45% (*p* < 0.001), and the GAG content was preserved at 117% (ns) and significantly increased by 17% (*p* = 0.022) (normalized to the total proteins) after decellularization, respectively (Pashos et al., 2017; Oganesyan et al., 2023). These differences in ECM preservation could be explained by the use of different detergents, such as the increase in GAG content due to the use of SDC or lower SDS concentration (Peloso et al., 2016; Alshaikh et al., 2019) and its expression after normalization to the total amount of proteins.

The reduction in GAG content can also explain the significant loss of total proteins, GFs, and cytokines, which are bound to the ECM by GAGs (Reing et al., 2010; 2010; Badylak et al., 2011; Crapo et al., 2011; Holgersson, 2016; Di Meglio et al., 2017; Duisit et al., 2017; 2018a). Despite that, after decellularization, we detected, in a lower amount than in control tissues, human growth factors and cytokines implicated in epithelialization (EGF, KGF, FGF, PDGF, HIF, and GMSCF), angiogenesis (VEGF, HGF, EGF, IGF, PDGF, HIF-1, and CXCL12), or cell recruitment (TGF-B1, TGFB2, GM-CSF, and PDGF) and promoting the matrix integration during wound repair (Barrientos et al., 2014; Su et al., 2014; Rodrigues et al., 2019; Shpichka et al., 2019). In addition, pre-loading the ECM with GFs allows their better release to seeded or adjacent cells, thus improving the recellularization, *in vivo* integration, and revascularization of acellular scaffolds (Loai et al., 2010; Lugo et al., 2011; Chen et al., 2014; Su et al., 2014; Liu et al., 2021).

Binding to collagen and elastin fibers, SDS can damage ECM architecture (Crapo et al., 2011; Keane et al., 2015), influence cell development (Rieder et al., 2004; Gratzer et al., 2006; Cebotari et al., 2010; Reing et al., 2010; Andrée et al., 2014; Zvarova et al., 2016; Alizadeh et al., 2019; Kraft et al., 2020), and promote *in vivo* host adverse reactions (Friedrich et al., 2018). Combining DIW and PBS bath steps, the residual SDS in the produced scaffolds was below different levels considered non-cytotoxic and similar to those found by previous research groups (Gratzer et al., 2006; Cebotari et al., 2010; Zvarova et al., 2016; Roderjan et al., 2019; Kraft et al., 2020). Associated with the increasing proliferation of the seeded fibroblasts, these results confirmed the non-toxicity of d-NAC scaffolds and their ability to promote cell adherence and proliferation.

Acellular scaffolds were implanted subcutaneously in rats for 30 days to assess their *in vivo* biocompatibility, revascularization, and integration by the host tissue, which will be critical for future clinical applications in reconstructive surgery. POD30 was chosen as the endpoint because it allows us to detect the generation of rat anti-donor IgG (Duisit et al., 2018a; 2018b) and the *in vivo* revascularization of the entire thickness of the scaffold (Eppley, 2000; Klar et al., 2014; Jank et al., 2017; Duisit et al., 2018a; Duisit et al., 2018b; Pashos et al., 2020; Caronna et al., 2021). Decellularized scaffolds were still observable and integrated with the surrounding tissue, showing an entire *in vivo* revascularization with a local inflammation, and were fully infiltrated by host cells, including 25% of immune cells. In comparison, implanted n-NACs presented a foreign body reaction associated with a thick fibrosis encapsulation containing immune cells and neo-vessels and were not infiltrated after more than 4 weeks of implantation, as also noted by other teams (Wilshaw et al., 2008; Jank et al., 2017). In addition to a well-known ECM remodeling and revascularization of the scaffolds by surrounding host tissues (Londono and Badylak, 2015), we observed a significantly lower infiltration of CD68-expressing cells (pan-macrophages) and a similar infiltration of CD3-expressing cells (pan-lymphocytes). This fact highlights that d-NAC scaffolds and their residual ECM proteins can support cellular migration and neoangiogenesis into the scaffold. The host immune reaction can be promoted by residual small cell debris or DNA fragments, but other components also seem to play an important role in immune regulation (Morwood and Nicholson, 2006; Somers et al., 2012; Keane et al., 2015). Indeed, inflammatory and immune responses can not only be influenced by the decellularization agents but can also be supported by residual molecules and cell remnants as damage-associated molecular patterns (DAMPs), mitochondrial residues, or ECM proteins (Zhang et al., 2010; Fishman et al., 2013; Keane et al., 2015). Furthermore, fragments of ECM proteins exposed by the decellularization process, as well as other minor ECM proteins called matricellular proteins, which are functional and pro-inflammatory rather than structural, can promote a strong inflammatory and immune response (Morwood and Nicholson, 2006; Kasravi et al., 2023). Nevertheless, scaffolds can also activate a lymphocyte T-reg and T-h2 response, which corresponds more to tissular remodeling, integration, and wound healing associated with an anti-inflammatory response, and is, thus, considered a graft acceptance rather than a trigger of tissue rejection (Allman et al., 2001; Hillebrandt et al., 2019; Yu et al., 2022). Despite this, the absence of rat anti-human IgG in the d-NAC group, in contrast to the production of IgG in all animals of the n-NAC group, confirmed the acceptance of the acellular graft (Duisit et al., 2018a).

As a potential preclinical model, we confirmed the ability of d-NACs to support cell adhesion and proliferation and to regenerate a cutaneous epithelium using the RHE technique (De Vuyst et al., 2013). Keratinocytes can be harvested from small skin biopsies and quickly expanded *in vitro* (Dragúňová et al., 2013) in order to be used with the RHE technique or to generate cultured autologous keratinocyte sheets (Vig et al., 2017). The use of hydrogels containing fibrinogen, fibronectin, or KGF could improve not only uniform cell delivery on the irregular dermis of the nipple *in vitro* but also the *in vivo* integration and revascularization of d-NAC scaffolds (Lugo et al., 2011). However, these *in vitro* improved regeneration techniques are time consuming, expensive, and not always efficient (Dragúňová et al., 2013; Vig et al., 2017). Alternatively, the d-NAC scaffold could be implanted *in vivo* as a 3D vector for secondary skin healing arising from the surrounding host skin, which is a safe biological and well-known process of tissue integration, highly studied since the use of the ADM in surgery (Wainwright, 1995; Eppley, 2000; Carruthers et al., 2015; Boháč et al., 2018; Gierek et al., 2022) and also highlighted after the *in vivo* implantation of the bioengineered dermis in an animal narrow skin defect model (Jank et al., 2017; Pashos et al., 2020; Caronna et al., 2021). Moreover, after the implantation of Alloderm^©^ as an areolar dermal onlay graft, the re-epithelialization lasts about 8.1 weeks (Rao et al., 2014). On a d-NAC scaffold, this long delay in skin surface restoration could result in tissue contraction and the loss of the 3D architecture of the complex. Therefore, from a clinical point of view, thin split-thickness skin grafts (STSGs), as routinely performed to cover commercial ADM (Wainwright, 1995; Dussoyer et al., 2020; Gierek et al., 2022; Petrie et al., 2022), should be considered a more straightforward, fast, efficient, and safe method to fully re-epithelialize the entire surface of the areola and the nipple relief. Keratinocyte sprays, as used in burn surgery, are also an option to reepithelialize the entire small NAC area (Horch et al., 2001; Kopp et al., 2004; Pleguezuelos-Beltrán et al., 2022). The specific pigmentation of the NAC will also be challenging. NAC skin contains 2 and 1.5 times more melanin and melanocytes, respectively, than breast skin (Dean et al., 2005). Pigmented RHE could be achieved *in vitro* using various melanocyte concentrations or phenotypes (Berking and Herlyn, 2023), which are durable after transplantation (Medalie et al., 1998; Berking and Herlyn, 2001). However, it seems difficult to regenerate the same contralateral NAC pigmentation. Tattooing after re-epithelialization seems ideal for the first clinical outcomes, even if it needs to be repeated.

Scaffold re-innervation will be a challenging process. Clinical studies applying neurotization to re-innerve the NAC after mastectomy showed an improved sensibility (Deptula and Nguyen, 2021; Tevlin et al., 2021). Despite the few reports of acellular matrix re-innervation, recent works identified, in parallel with the *in vivo* scaffold revascularization, neo-nerves in acellular pericardial (Gálvez-Montón et al., 2015) scaffolds and acellular nipple (Caronna et al., 2021) scaffolds after 30 days and 6 weeks of implantation, respectively, confirming the ability of such decellularized ECMs to be integrated into host tissues and support neural cell migration and differentiation. In addition, the acellular nerve ECM also promotes axon migration through the residual nerve scaffold (de Luca et al., 2014). The preserved acellular nerve ramifications identified in decellularized scaffolds could be recolonized after implantation by the host subcutaneous sensitive nerves throughout a process of neighborhood neurotization and, consequently, could then improve sensory recovery (Guo et al., 2013; Gálvez-Montón et al., 2015; Caronna et al., 2021).

NAC tissue engineering allows us to consider its preclinical application according to two clinical approaches. The first approach is creating NAC scaffold biobanks with precise donor morphological pre- and post-decellularization characteristics to offer the best match with the recipient patient’s NAC. Cryopreservation, already used in biobanking (Jashari, 2021), seems to be the best solution for preserving structural, mechanical, and cellular tissue properties (Theodoridis et al., 2016; Iop et al., 2017; Urbani et al., 2017; Zouhair et al., 2019). Another approach could be relying on the decellularization and banking of the patient’s own NAC, harvested from her mastectomy specimen. This *in vitro* step would allow us to remove all potential cancer cells and provide, thereafter, a bioengineered graft matching perfectly with the contralateral and initial NAC. However, in the case of R1 resection, and even in the case of R0 resection, studies should be led to refute the neoplastic potential of this ECM scaffold. Indeed, it has been recently highlighted that, compared to healthy tissues, (pre-)tumoral acellular ECMs have a modified microenvironment, both in terms of structure and molecular composition (over or lower expression of ECM proteins, GFs, and cytokines). Thus, the tumoral ECM can alter by itself the cellular metabolism and promote cell growth, proliferation, modification in cell phenotypes as well as the vascular network formation, with all of those biological phenomena leading in potential tumorigenesis and the development of cancer (Miyauchi et al., 2017; Romero-López et al., 2017; Piccoli et al., 2018; Mazza et al., 2019; García-Gareta et al., 2022; Gentilin et al., 2022). If demonstrated to be oncologically safe and surgically reliable, such a bioengineered NAC autograft procedure should be the ultimate achievement of personalized breast reconstructive surgery.

## 5 Conclusion

Tissue engineering techniques allow to create acellular and biocompatible NAC scaffolds with a preserved specific 3D morphology, microarchitecture, and matrix proteins. Decellularized NAC scaffolds preserve their cell growth potential by supporting cell adhesion, proliferation, and migration. They also retain their ability to regenerate a skin epidermis *in vitro* and to be *in vivo* revascularized and integrated by host surrounding tissue. However, additional *in vivo* animal studies are required before the first clinical applications to assess the long-term nipple morphology retention and integration after implantation. Moreover, it will be necessary to evaluate the best way to epithelialize and pigment the NAC scaffold, recover nipple sensibility, and implant the scaffold on the recipient breast, in terms of costs, risks, and long-term patient’s satisfaction.

## Figures and Tables

**Table:** 

**3D**	Three dimensional
**ADM**	Acellular dermal matrix
**DAB**	3,3′-Diaminobenzidine
**DAPI**	4′,6-Diamidino-2-phenylindole
**DIW**	Deionized water
**DNA**	Deoxyribonucleic acid
**DRP**	Decellularization and recellularization process
**ECL**	Enhanced chemiluminescence
**ECM**	Extracellular matrix
**FBS**	Fetal bovine serum
**FCM**	Fibroblast culture medium
**GAGs**	Glycosaminoglycans
**GFs**	Growth factors
**H&E**	Hematoxylin and eosin
**HEKa**	Human keratinocytes
**HFs**	Human fibroblasts
**HLA**	Human leukocyte antigen
**HRP**	Horseradish peroxidase
**IF**	Immunofluorescence
**IHC**	Immunohistochemistry
**KCM**	Keratinocyte culture medium
**MBAS**	Methylene Blue Active Substance Assay
**MHC**	Major histocompatibility complex
**MT**	Masson’s trichrome
**NAC**	Nipple–areolar complex
**P/S**	Penicillin/streptomycin
**PBMCs**	Peripheral blood mononuclear cells
**PBS**	Phosphate-buffered saline
**POD**	Post-operative day
**RHE**	Reconstructed human epidermis
**rpm**	Rotation per minute
**RT**	Room temperature
**SD**	Standard deviation
**SDS**	Sodium dodecyl sulfate

## Data Availability

The original contributions presented in the study are included in the article/Supplementary Material, further inquiries can be directed to the corresponding author.
